# Pulmonary Nodule Detection in Patients with a Primary Malignancy Using Hybrid PET/MRI: Is There Value in Adding Contrast-Enhanced MR Imaging?

**DOI:** 10.1371/journal.pone.0129660

**Published:** 2015-06-11

**Authors:** Kyung Hee Lee, Chang Min Park, Sang Min Lee, Jeong Min Lee, Jeong Yeon Cho, Jin Chul Paeng, Su Yeon Ahn, Jin Mo Goo

**Affiliations:** 1 Department of Radiology, Seoul National University College of Medicine, and Institute of Radiation Medicine, Seoul National University Medical Research Center, Seoul, Korea; 2 Cancer Research Institute, Seoul National University, Seoul, Korea; 3 Department of Radiology, Duke University Medical Center, Durham, North Carolina, United States of America; 4 Department of Nuclear Medicine, Seoul National University College of Medicine, Seoul, Korea; National Cancer Center, JAPAN

## Abstract

**Purpose:**

To investigate the added value of post-contrast VIBE (volumetric-interpolated breath-hold examination) to PET/MR imaging for pulmonary nodule detection in patients with primary malignancies.

**Materials and Methods:**

This retrospective institutional review board–approved study, with waiver of informed consent, included 51 consecutive patients who underwent ^18^F-fluorodeoxyglucose (FDG) PET/MR followed by PET/CT for cancer staging. In all patients, the thorax was examined with pre-and post-contrast VIBE MR with simultaneous PET acquisition. Two readers blinded to the patients’ data independently recorded their level of suspicion for pulmonary nodules based on PET, pre-contrast VIBE, and fused PET/MR images (first session), and reassessed them 4-weeks later after addition of post-contrast VIBE (second session). Jackknife alternative free-response receiver-operating-characteristic (JAFROC) analysis was performed, with PET/CT as the reference standard.

**Results:**

A total of 151 pulmonary nodules (44 FDG-avid, 107 non-FDG-avid nodules) were detected on PET/CT, including 62 nodules≥5mm in diameter and 89 nodules<5mm. In the first session, the average nodule detection rate was 53.3% for all nodules, 97.7% for FDG-avid, 35.0% for non-FDG-avid nodules, 87.9% for nodules≥5mm and 29.2% for nodules<5mm. In the second session, the average detection rate was 53.3% for all nodules, 97.7% for FDG-avid, 35.0% for non-FDG-avid nodules, 85.5% for nodules≥5mm and 30.9% for nodules<5mm. The average JAFROC figure-of-merit was 0.837 in the first session and 0.848 in the second session. There were no significant differences in detection performance between sessions (P=0.48).

**Conclusion:**

The addition of post-contrast VIBE to hybrid PET/MR imaging provided no additional value in the detection of pulmonary nodules.

## Introduction

The development of the hybrid PET/MRI system is a milestone in radiology and nuclear medicine, one of the most potent clinical applications of which is oncologic imaging [1–4]. Although PET/CT has become widely accepted in many hospitals as a part of their routine clinical practice for various cancers [5–9], with PET/MRI, excellent soft-tissue contrast resolution and functional imaging data from specialized MR techniques in assessing tumor burden can be obtained [10]. Moreover, PET/MRI contributes to radiation dose reduction, which is particularly of interest in oncologic patients who undergo multiple repeated examinations. In addition, the safety profile of MR contrast agents as well as the availability of some targeted MR contrast media has been shown to be appealing compared with CT contrast agents [4].

However, there is a limitation in the detection performance of the MRI portion of PET/MR for pulmonary nodules, which has been recognized as one of the major demerits of hybrid PET/MRI imaging [11–13]. This is particularly important for oncologic patients, in whom accurate detection and characterization of pulmonary nodules can be critical for their management. With CT, even small pulmonary nodules less than 5 mm can be easily identified owing to its excellent contrast resolution in the lung parenchyma, even without the injection of intravenous contrast media [13]. On the other hand, the role of MR imaging for pulmonary imaging has been limited. Recently, the availability of high performance gradient systems and innovative sequences with parallel imaging has introduced new, promising approaches to pulmonary imaging with MRI [14]. Today, fast T1-weighted gradient-echo (GRE) sequences can depict pulmonary nodules in the range of 3–5 mm [14]. Furthermore, with the hybrid PET/MRI system, even better performance in pulmonary nodule detection has been demonstrated than MRI alone owing to the additional metabolic information of FDG uptake [10]. In this previous study, however, low sensitivity for small non-FDG avid nodules was reported using PET/MRI and a pre-contrast radial VIBE (volumetric interpolated breath-hold examination) sequence for the detection of pulmonary nodules [10]. As MR contrast agents can enhance vascular structures and better clarify the anatomic location, we hypothesized that there may be a complementary role for MR contrast agents in PET/MRI for pulmonary nodule detection.

Therefore, the purpose of our study was to investigate the added value of a post-contrast VIBE sequence to hybrid PET/MR imaging for the detection of pulmonary nodules in patients with primary malignancies.

## Materials and Methods

This retrospective study received institutional review board approval and the requirement for additional informed consent was waived.

### Study Population

The study population in this study was collected from on-going prospective research cohorts at our institution with full consent from the principal investigators, and the study purpose and methodology of the present study was completely different from those studies (unpublished) which investigated the performance of hybrid PET/MRI in cancer staging. Between March 2013 and Feb 2014, 646 patients with primary malignancies underwent ^18^F-fluorodeoxyglucose (FDG) PET/MR imaging for research purposes at our institution. Among them, 51 consecutive patients (a) who underwent PET/CT imaging on the same day and (b) who underwent thoracic imaging using pre- and post-contrast VIBE sequences for PET/MRI were included in this study. These fifty-one patients with a mean age of 63.3 years and an age range of 35–84 years (34 men; mean age, 64 years; age range, 35–84 years; 17 women; mean age, 61.8 years; age range, 40–80 years) constituted our study population. The underlying primary malignancies of our study population were as follows: lung cancer (n = 43), pancreatic cancer (n = 7) and prostate cancer (n = 1).

### Hybrid PET/MR Imaging Technique

All patients had fasted for at least 6 hours prior to intravenous administration of ^18^F-FDG (5.2 MBq/kg of body weight). Serum glucose levels were checked with a blood glucose meter prior to the injection and were less than 200 mg/dl in all patients. After administration, patients were rested for 60 minutes prior to imaging. PET/MR exams were performed using a whole-body hybrid PET/MR system (Biograph mMR; Siemens Healthcare, Erlangen, Germany), in which simultaneous acquisition of PET and MR data were obtained.

The scan range of whole body oncologic PET/MR was from the head to the mid-thigh using 5 or 6 bed positions. In each bed position, a coronal 3D VIBE sequence for Dixon-based attenuation correction was first acquired. Thereafter, patients underwent different MR scan protocols as per their primary malignancies, simultaneously with PET scanning. In our study, all patients underwent pre- and post-contrast 3D axial T1-weighted gradient-echo (VIBE) sequences covering the whole body with the thorax examined with a breath hold. The post-contrast axial 3D VIBE sequence was obtained with a delay of 4 minutes using a dose of 0.1mmol/kg of gadoteric acid (Dotarem; Guerbet, Aulnay-sous-Bois, France) in lung cancer (n = 43) and prostate cancer (n = 1) patients. The injection rate was 3 mL/sec, followed by a 20 mL bolus of saline at the same rate. In the seven pancreatic cancer patients, the post-contrast axial 3D VIBE sequence was obtained using a total dose of 15 mL of gadoxetic acid disodium (Primovist; Bayer-Schering, Berlin, Germany) with a delay of 10 minutes after the 2nd injection (1st injection, 5 mL with an injection rate of 3 mL/sec; 2nd injection performed 5 minutes later, 10mL with an injection rate of 1.5 mL/sec). Axial 3D VIBE sequences were acquired with the following parameters: repetition time/echo time, 3.4msec/1.22msec; section thickness, 3.0 mm; flip angle, 9°; 80 axial slices; bandwidth, 540 Hz per pixel; voxel size, 1.6 x 1.2 x 3.0 mm; quick fat-saturation mode; generalized autocalibrating partially parallel acquisitions factor, two; and number of sections per slab, 72. The additional acquisition time for the post-contrast axial 3D VIBE sequence covering the whole body was approximately 5 minutes. PET data were corrected for attenuation and reconstructed on a 172 × 172 matrix using a Gaussian filter with 6.0 mm full width at half maximum, and a three-dimensional ordered-subsets expectation maximization algorithm (iterations, three; subsets, 21; zoom of 1.0).

### PET/CT Technique

PET/CT was acquired after completion of PET/MR scanning with a 120 minute delay from the prior ^18^F FDG injection. Whole body PET images were performed with the conventional protocol of ^18^F-FDG PET using Biograph 40 (Siemens Medical Solutions, Knoxville, TN). CT images were acquired from the head to the mid-thigh using a tube voltage of 120 kVp, tube current of 40 mA, tube-rotation time of 0.75 s per rotation, and a pitch of 1.5. CT images were reconstructed with 5 mm thickness. Immediately after CT, emission PET images were acquired for 2 minutes per each bed using the three-dimensional acquisition mode. The resulting PET and CT images were co-registered on hardware.

### Image Analysis

#### PET/CT

One unblinded board-certified radiologist (K.H.L., with 6 years of experience in general imaging) and one unblinded board-certified nuclear medicine physician (J.C.P., with 14 years of experience in nuclear medicine) analyzed the PET/CT images to set the reference standards of pulmonary nodules. For the information of number, location and size of the nodules, K.H.L. identified all visible pulmonary nodules on axial CT images and measured the longest diameters of the nodules on the axial plane. To determine FDG-avidity, J.C.P. classified the nodules as FDG-avid when the activity was greater than the background activity in the surrounding tissue and unrelated to the physiologic tracer uptake [15] and measured the maximum standardized uptake value (SUV_max_) of all FDG-avid nodules at least 1 cm in size at PET/CT. These data served as the reference standards for the visibility analysis and nodule detection performance on PET/MRI. Calcified nodules were excluded from the analysis as they are known to be devoid of an MR signal.

#### PET/MRI

One unblinded board-certified nuclear medicine physician (J.C.P.) determined the FDG avidity and measured the SUV_max_ of all FDG-avid nodules at least 1cm in size at PET/MRI. FDG avidity was identified when the activity was greater than the background activity in the surrounding tissue and unrelated to the physiologic tracer uptake [15].

Visibility analysis was performed by one radiologist (S.M.L., with 5 years of experience in thoracic imaging), who retrospectively graded the visibility of the corresponding nodules on PET and MR images for all visible nodules on PET/CT. On PET images, the visibility of nodules was determined as visible or non-visible. On MR images, the visibility of nodules was evaluated based on a five-point scale (0–4). A score of 4 represented the highest degree of visibility (definitely visible) and a score of 0 represented non-visibility. Nodule visibility evaluation on MR images was separately performed on pre-contrast VIBE and post-contrast VIBE MR sequences.

To evaluate the nodule detection performance of PET/MRI, two chest radiologists (J.M.G. with 25 years of experience and C.M.P. with 14 years of experience in thoracic imaging) who were blinded to all clinical and histopathologic data retrospectively and independently interpreted the PET/MR imaging studies. Reader 1 (J.M.G.) and Reader 2 (C.M.P.) both had at least 1 year of experience in interpreting PET/MR images. The readers performed two separate reading sessions with a 4-week interval between readings to minimize recall bias. During the first session, readers initially reviewed the PET images alone and annotated the FDG-avid nodules. Thereafter, pre-contrast axial 3D VIBE and axial fused PET/MR images were provided together along with the PET images. Readers were instructed to mark all detected nodules along with their confidence score on pre-contrast VIBE images. In the second session, PET data were reviewed first and then PET, axial pre-contrast 3D VIBE, axial fused PET/MR, and axial post-contrast 3D VIBE images were provided together. Readers were instructed to mark all detected nodules along with their confidence score on post-contrast VIBE images. All images were interpreted at a picture archiving and communication system (PACS) workstation (Infinitt Co, Ltd, Seoul, Korea). On PET imaging, only the presence or absence of FDG-avid nodules was determined. On MR imaging, the readers were instructed to mark all detected nodules of any size along with their confidence scores as follows: 5, definitely a nodule; 4, probably a nodule; 3, possibly a nodule; 2, unlikely to be a nodule; and 1, very unlikely to be a nodule [16]. The readers were also asked to mark each detected nodule on a hardcopy film to avoid miscounting.

Finally, one unblinded researcher (K.H.L.) collected all marks and confidence levels and performed a nodule-by-nodule comparison in each patient by comparing PET/MR images with PET/CT images (reference standard). For each nodule on a PET/CT image, the detection of that nodule on PET and MR images was noted. If the nodule present on a PET/CT image was not annotated by the readers on a particular PET or MR image, it was considered as a missed nodule [10].

### Statistical Analysis

Visibility scores of nodules on both pre-contrast and post-contrast VIBE images were compared using the Wilcoxon test. Reader agreement for nodule detection on PET/MR imaging was assessed in terms of weighted kappa coefficients. Nodule detection performance of the two readers on PET/MR imaging was evaluated using nodule detection rates and figures of merit (FOMs). The nodule detection rate was calculated as the proportion of detected true-positive nodules, regardless of their confidence score. The nodule detection rates were computed for PET alone, PET/MR images without the post-contrast VIBE sequence, and PET/MR with the post-contrast VIBE sequence, and compared using the McNemar test. Nodule detection rates stratified by FDG avidity and nodule size were also calculated. FOMs were calculated using Jackknife FROC software (JAFROC, version 4.2; http://www.devchakraborty.com) [17, 18], specifically developed to analyze observer free-response tasks [17, 18]. An FOM is defined as the probability that lesions including unmarked lesions are rated higher than non-lesion marks on control [18], or in other words, the probability that lesions are given a higher confidence rating for the presence of nodules than normal findings. Both normal images with no marks and unmarked lesions were assigned a zero rating. For all studies, a difference with a *P* value of less than 0.05 was considered to indicate a statistical significance.

## Results

On PET/CT, a total of 151 pulmonary nodules were detected including 44 FDG-avid and 107 non-FDG-avid nodules. Sixty-two nodules were 5 mm or larger in diameter and 89 nodules were small than 5 mm in diameter. All 44 FDG-avid nodules on PET/CT were ≥ 5 mm in diameter and also showed FDG-avidity on PET/MRI. Means ± standard deviations of the SUV_max_ of FDG-avid nodules were 12.8 ± 7.5 (range, 2.1–35.3) on PET/CT and 8.8 ± 5.2 (range, 1.2–21.4) on PET/MRI, respectively.

### Visibility of Nodules on PET/MR Imaging

**Fig 1:**
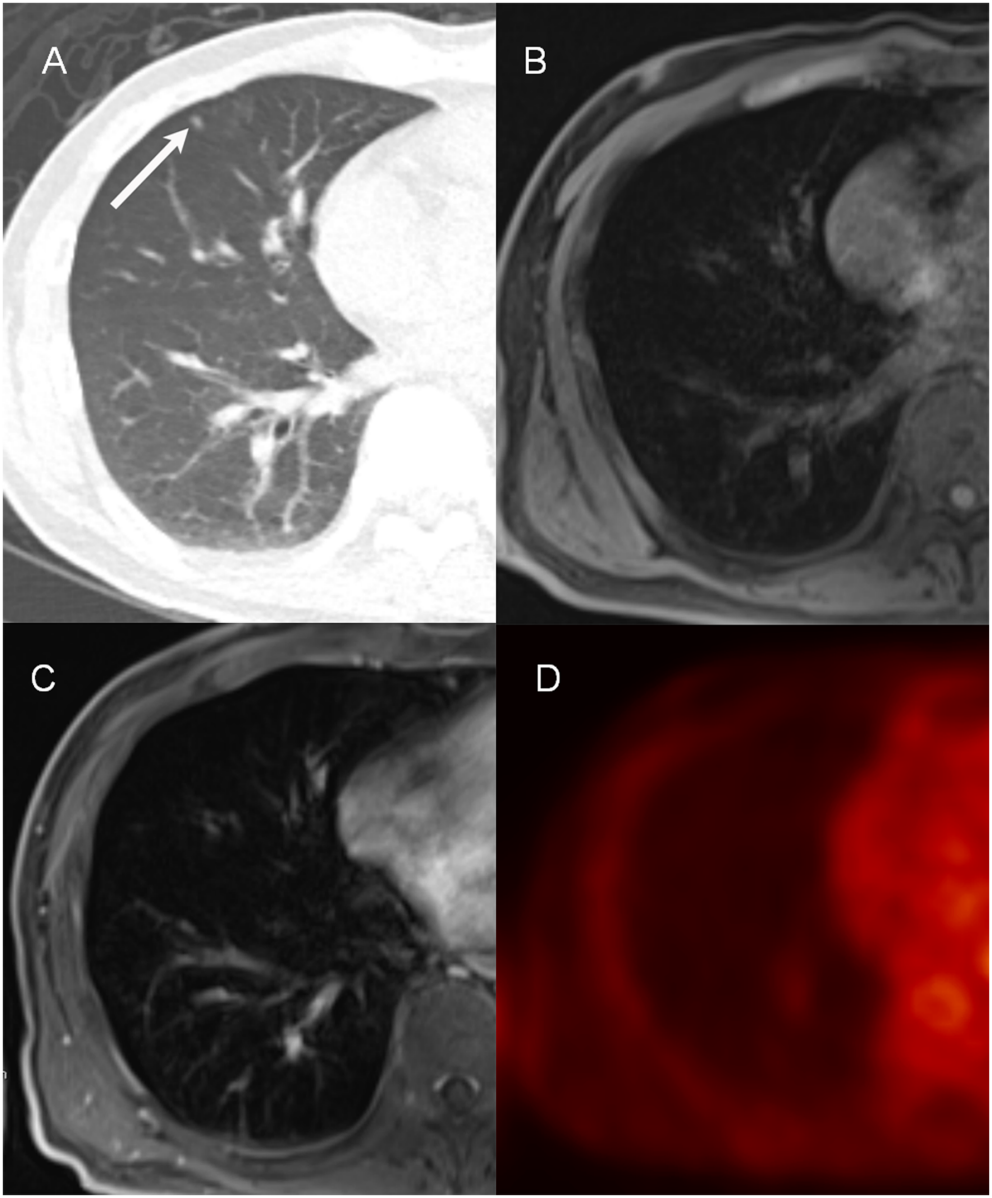
An example of the invisible nodule on PET/MR images (57-year-old man with pancreatic cancer). A tiny nodule (3 mm) is identified (arrow) in the right middle lobe on the CT image (A). This lesion is not visible on pre-contrast VIBE (B) and post-contrast VIBE (C) images. None of the readers were able to identify this nodule during the first and second sessions. This lesion is not visible on PET (D) images due to non-FDG-avidity.

The proportions of visible nodules on PET and MR images using pre-contrast VIBE and post-contrast VIBE sequences are shown in Table 1. Almost all invisible nodules on PET/MR images (30/30 nodules on pre-contrast VIBE and 25/26 nodules on post-contrast VIBE) were < 5 mm in diameter (Fig 1), except one nodule 6 mm in diameter which was not visible on the post-contrast VIBE image. The median visibility score was 2 (interquartile range, 1–4) on both pre-contrast and post-contrast VIBE images. There were no significant differences in visibility scores of nodules on pre-contrast and post-contrast VIBE images (*P* = 0.33).

**Table 1:** Proportion of Visible Nodules on PET and MR Images with Pre- and Post-contrast VIBE Sequences.

	PET Images	MR (Pre-contrast VIBE)	MR (Post-contrast VIBE)
All	29.1 (44/151) [22.0–37.0]	80.1 (121/151) [72.8–86.1]	82.8 (125/151) [75.8–88.4]
FDG avid	100 (44/44) [92.0–100]	100 (44/44) [92.0–100]	100 (44/44) [92.0–100]
Not FDG avid	0 (0/107) [0–3.4]	72 (77/107) [62.5–80.3]	75.7 (81/107) [66.5–83.5]
≥5mm in diameter	71 (44/62) [58.1–81.8]	100 (62/62) [94.2–100]	98.4 (61/62) [91.4–100]
<5mm in diameter	0 (0/89) [0–4.1]	66.3 (59/89) [55.5–76.0]	71.9 (64/89) [61.4–80.9]

Note.—Data in parentheses are the raw data used to calculate percentages. Data in brackets are 95% confidence intervals.

### Nodule Detection Performance on PET/MR Imaging

**Fig 2:**
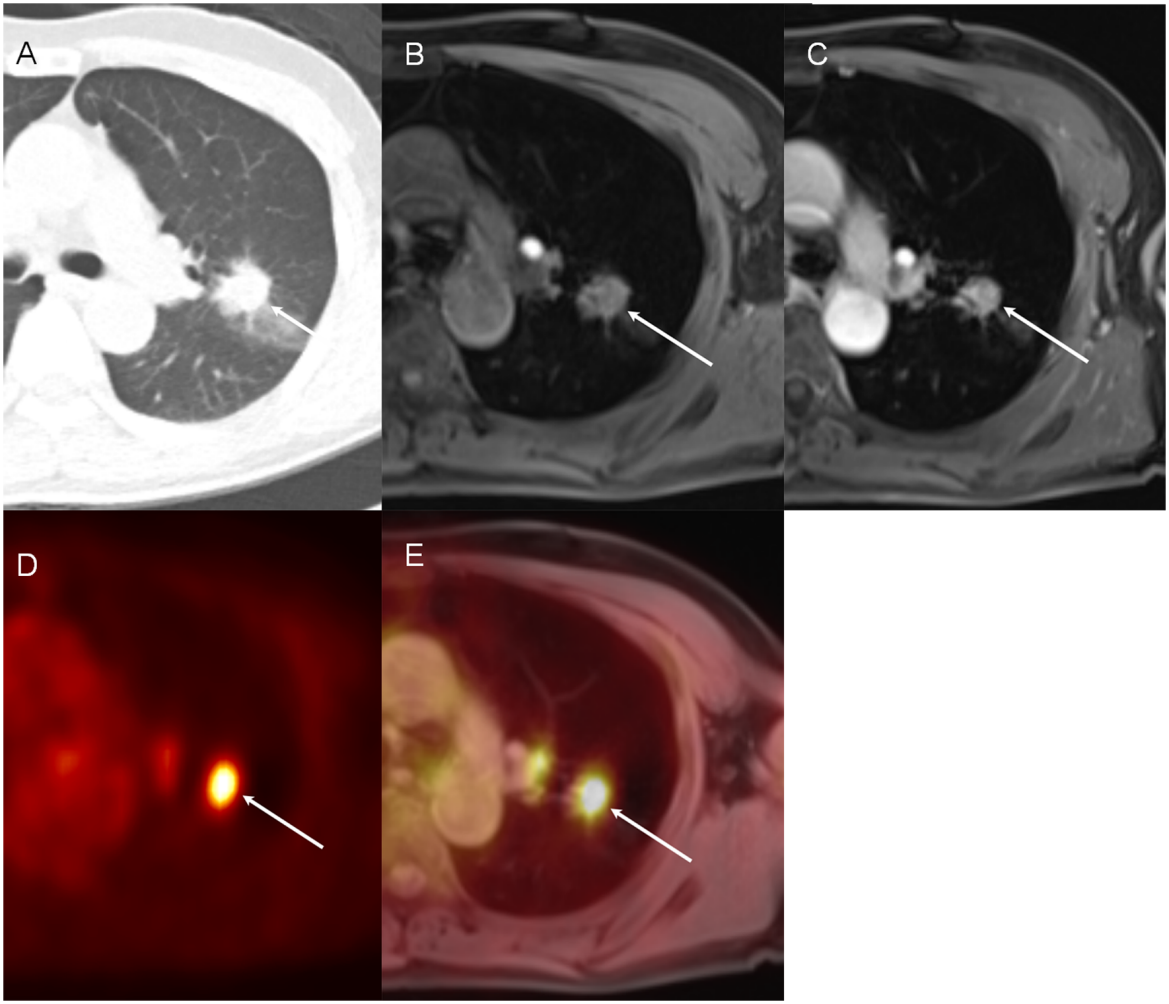
An example of the FDG-avid nodule on PET/MR images (67-year-old man with lung cancer). The primary lung cancer lesion (arrow) is seen on CT image (A), pre-contrast VIBE (B) and post-contrast VIBE (C) images. This nodule (arrow) is also well-delineated as an FDG-avid nodule in the left upper lobe on PET (D) and fused PET/MR images (E).

**Fig 3:**
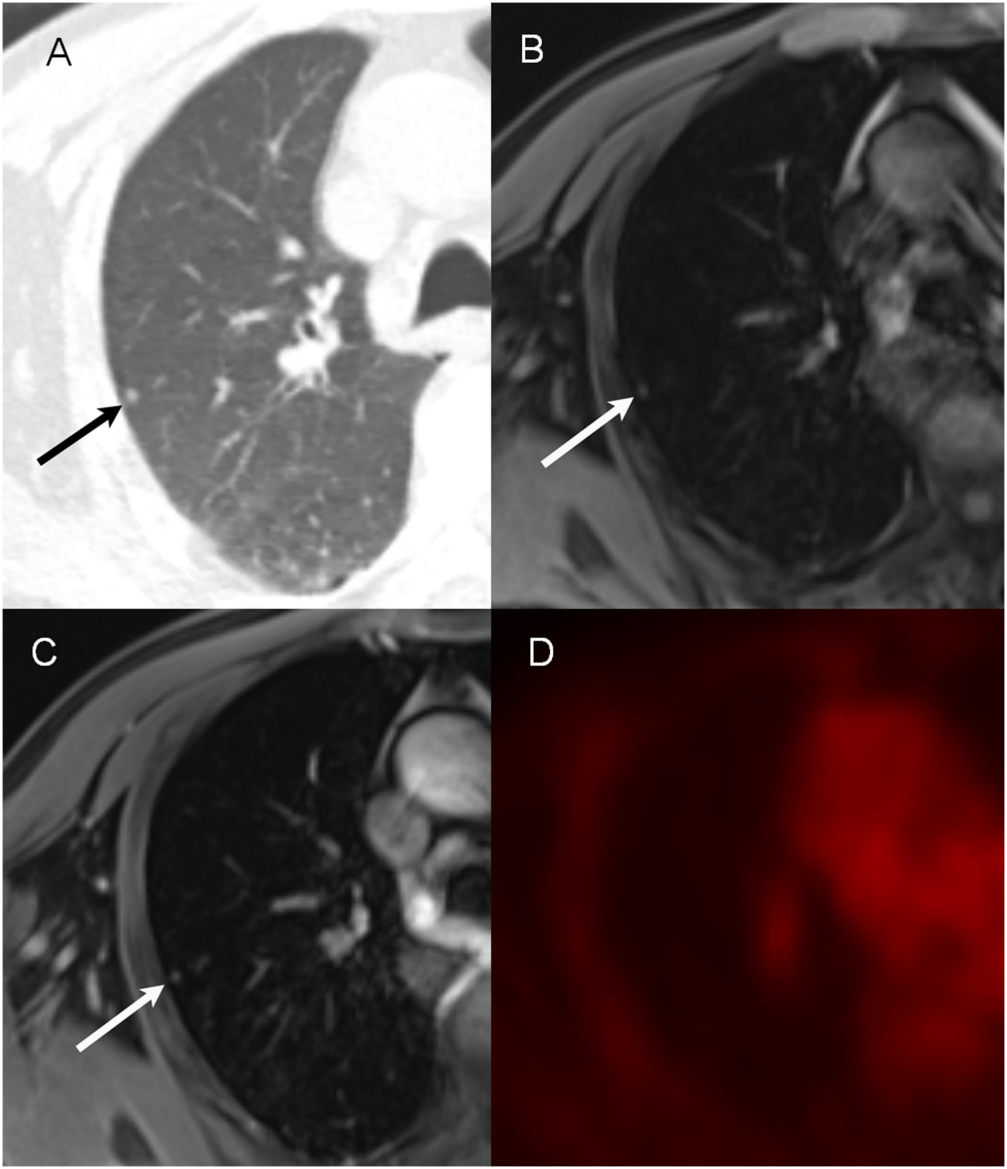
An example of the non-FDG-avid nodule on PET/MR images (68-year-old man with lung cancer). On CT image (A), a tiny nodule (3 mm) is identified (arrow) in the right upper lobe. This lesion is also visible on pre-contrast VIBE (B) and post-contrast VIBE (C) images. Both readers identified this nodule during both the first and second sessions. However, this lesion is not visible on PET (D) images due to non-FDG-avidity.

Readers demonstrated substantial agreement in nodule detection (weighted kappa = 1.00 for PET, weighted kappa = 0.81–82 for PET/MR). Nodule detection rates on PET images and PET/MR images during the first and the second sessions are shown in Table 2. All 43 patients with FDG-avid nodules were identified on PET images (Fig 2). PET had a sensitivity of 28.5% (43/151) for all nodules and a sensitivity of 97.7% (43/44) for FDG-avid nodules. Both readers missed one FDG-avid nodule measuring 11 mm in diameter with low-level FDG uptake (SUV_max_, 1.1 on PET/MR images). PET did not depict any nodules that were non-FDG-avid or < 5 mm in diameter. To the contrary, PET/MR images enabled the detection of non-FDG-avid nodules and small nodules < 5 mm in diameter. In the first session, the average nodule detection rate was 53.3% for all nodules, 97.7% for FDG-avid nodules, 35.0% for non-FDG-avid nodules, 87.9% for nodules ≥ 5 mm in diameter and 29.2% for nodules < 5 mm in diameter. In the second session with the addition of a post-contrast VIBE sequence, the average nodule detection rate was 53.3% for all nodules, 97.7% for FDG-avid nodules, 35.0% for non-FDG-avid nodules, 85.5% for nodules ≥ 5 mm in diameter and 30.9% for nodules < 5 mm in diameter. PET/MR imaging improved the sensitivity for nodule detection compared with PET alone with significantly higher sensitivity for all nodules (53.3% vs 28.5%, *P* < 0.001), for nodules that were not FDG avid (35.0% vs 0%, *P* < 0.001), and for nodules that were < 5 mm in diameter (29.2–30.9% vs 0%, *P* < 0.001). However, there were no significant differences in nodule detection rates between the first and second sessions for both readers 1 and 2 (*P* = 0.86) (Fig 3). In addition, the numbers of false-positive nodules annotated by readers 1 and 2 were as follows: 41 and 8 false-positive nodules by readers 1 and 2 during the first session; 43 and 7 false-positive nodules by readers 1 and 2 during the second session, respectively.

**Table 2:** Comparison of Nodule Detection Rates on PET and PET/MR Images during the first (without post-contrast VIBE) and second sessions (with post-contrast VIBE).

	PET Images	PET/MR Images (First session)	PET/MR Images (Second session)
**Reader 1**			
All	28.5 (43/151) [21.5–36.4]	53.6 (81/151) [45.3–61.7]	52.3 (79/151) [44.0–60.5]
FDG avid	97.7 (43/44) [87.9–99.9]	97.7 (43/44) [87.9–99.9]	97.7 (43/44) [87.9–99.9]
Not FDG avid	0 (0/107) [0–3.4]	35.5 (38/107) [26.5–45.3]	33.6 (36/107) [24.8–43.4]
≥5mm in diameter	69.4 (43/62) [56.4–80.5]	91.9 (57/62) [82.1–97.3]	83.9 (52/62) [72.4–92.0]
<5mm in diameter	0 (0/89) [0–4.1]	27.0 (24/89) [18.1–37.5]	30.3 (27/89) [21.0–41.0]
**Reader 2**			
All	28.5 (43/151) [21.5–36.4]	53 (80/151) [44.7–61.2]	54.3 (82/151) [46.0, 62.4]
FDG avid	97.7 (43/44) [87.9–99.9]	97.7 (43/44) [87.9–99.9]	97.7 (43/44) [87.9–99.9]
Not FDG avid	0 (0/107) [0–3.4]	34.6 (37/107) [25.7–44.4]	36.4 (39/107) [27.3–46.3]
≥5mm in diameter	69.4 (43/62) [56.4–80.5]	83.9 (52/62) [72.4–92.0]	87.1 (54/62) [76.2–94.3]
<5mm in diameter	0 (0/89) [0–4.1]	31.5 (28/89) [22.1–42.2]	31.5 (28/89) [22.1–42.2]

Note.—Data in parentheses are the raw data used to calculate percentages. Data in brackets are 95% confidence intervals.

FOMs for PET/MR images during the first and second sessions are shown in Table 3. In the first session, the average FOM was 0.837 for all nodules, 0.984 for FDG-avid nodules, 0.776 for non-FDG-avid nodules, 0.952 for nodules ≥ 5 mm in diameter and 0.756 for nodules < 5 mm in diameter. In the second session, the average FOM was 0.848 for all nodules, 0.989 for FDG-avid nodules, 0.791 for non-FDG-avid nodules, 0.945 for nodules ≥ 5 mm in diameter and 0.781 for nodules < 5 mm in diameter. There were no significant differences in FOM between the first and second sessions (*P* = 0.48).

**Table 3:** Comparison of FOMs on PET/MR Images during the first (without post-contrast VIBE) and second sessions (with post-contrast VIBE).

	First PET/MR session (without post-contrast VIBE)	Second PET/MR session (with post-contrast VIBE)	*P-*value
**Reader 1**			
All	0.750 (0.668–0.833)	0.752 (0.664–0.841)	0.97
FDG avid	0.980 (0.951–1.00)	0.980 (0.952–1.00)	0.98
Not FDG avid	0.656 (0.553–0.759)	0.659 (0.545–0.772)	0.97
≥5mm in diameter	0.935 (0.891–0.979)	0.906 (0.844–0.968)	0.26
<5mm in diameter	0.622 (0.507–0.737)	0.645 (0.530–0.760)	0.77
**Reader 2**			
All	0.923 (0.868–0.977)	0.945 (0.897–0.993)	0.50
FDG avid	0.987 (0.966–1.00)	0.998 (0.993–1.00)	0.30
Not FDG avid	0.896 (0.827–0.966)	0.923 (0.858–0.987)	0.54
≥5mm in diameter	0.968 (0.938–0.998)	0.984 (0.967–1.00)	0.27
<5mm in diameter	0.891 (0.8167–0.966)	0.917 (0.846–0.987)	0.58

Note.—Data in parentheses are 95% confidence intervals. P values were calculated with Jackknife alternative free-response receiver-operating characteristic to compare figures of merit (FOM) on PET/MR images during the first and second sessions.

### Follow-up of False-negative and False-positive Nodules on PET/MR Imaging

A total of 94 nodules in 30 patients were missed by either reader 1 or 2. Among the 94 missed nodules, 93 nodules were non-FDG-avid and 80 nodules were < 5 mm in diameter. Follow-up was available for 82 nodules in 26 patients. The average follow-up period was 11 months (range, 5–18 months) with PET/CT (n = 1) and chest CT (n = 25). Among the 82 missed nodules on follow-up images, 20 nodules were pathologically confirmed as benign through surgery and 59 nodules were considered benign through follow-up clinical interpretation (53 nodules, stable; 6 nodules, disappeared). The interval changes of three nodules were not able to be evaluated as pneumonia or radiation pneumonitis had developed and the nodules were not identifiable. None of the missed nodules on PET/MR images were later confirmed as malignancies over follow-up.

A total of 86 false-positive nodules in 35 patients were marked by either reader 1 or reader 2. Among them, follow-up information was available for 82 false-positive nodules in 31 patients. The average follow-up period was 12 months (range, 3–18 months) with PET/CT (n = 3) and chest CT (n = 28). Among the 82 false-positive lesions, 20 lesions were not evaluable on follow-up studies as the patients had undergone same lobe lobectomy (n = 16) or developed pneumonia or radiation pneumonitis (n = 4). None of the 62 false-positive lesions on PET/MR images were later confirmed as true-positive nodules on follow-up studies.

## Discussion

In this study, we found that the application of contrast media provided no additional diagnostic advantage in the detection of pulmonary nodules on PET/MR imaging; the average FOM values showed no significant differences with the addition of contrast media (0.837 without the post-contrast VIBE sequence and 0.848 with the post-contrast VIBE sequence [*P* = 0.48]). Although the combination of PET and MR imaging in the hybrid PET/MR system in fact, enabled the detection of significantly more non-FDG-avid nodules (35.0% vs 0%, *P* < 0.001) and small nodules < 5 mm in diameter (29.2–30.9% vs 0%, *P* < 0.001) when compared with PET imaging alone, nodule detection rates were still low (35.0% for non-FDG-avid nodules and 30.9% for nodules < 5 mm, respectively), even with the addition of contrast-enhanced MR images.

Although MR contrast agents are known to be safer than CT contrast agents, the application of MR contrast media requires patient intravenous catheter insertion, increases the examination time, medical cost, and sometimes is accompanied by serious side effects including nephrogenic systemic fibrosis [19, 20]. Therefore, prior to its use, clear scientific evidence and rationale behind the use of MR contrast media must be provided. However, recent papers dealing with pulmonary nodule detection using the PET/MR system have adopted various protocols across studies, with [21] or without [10, 15] contrast media, as no guideline or consensus exists with regard to thoracic imaging using the PET/MR system. To our knowledge, this is the first report to evaluate and compare the nodule detection performance of pre- and post-contrast MR images in the hybrid PET/MR system, and according to our results, the addition of contrast-enhanced VIBE did not deliver a diagnostic advantage in pulmonary nodule detection. Thus, it seems that there is no need to routinely acquire a post-contrast VIBE sequence for the purpose of pulmonary nodule detection in the PET/MRI system. Yet, as contrast-enhanced MR imaging in oncologic PET/MR exam can still provide clinical value in lesion characterization, evaluation of local invasion by intra-thoracic tumors, tumor perfusion, or the detection of extrapulmonary metastasis, further investigations are warranted to establish the role of contrast-enhanced imaging when using PET/MRI.

Among the diverse MR sequences, 3D T1-weighted spoiled GRE sequence is the most frequently used sequence in thoracic imaging with the PET/MR system [10, 14, 15, 21, 22] and has previously been proposed in the literature [23] for chest MR imaging. In addition, the axial T1 GRE sequence is already used to scan the whole lung, abdomen and pelvis and is incorporated in the whole-body and partial-body PET/MRI protocols of many primary malignancies as it requires only 5 additional minutes [10, 24, 25]. However, in this study, we did not evaluate dedicated chest MR sequences including diffusion-weighted imaging, high-spatial-resolution half-Fourier acquisition single-shot turbo spin-echo or T2-weighted imaging with respiratory or cardiac gating, as the goal of this study was to evaluate the additional gain of post-contrast images as part of a routinely performed PET/MRI scan protocol of primary malignancies including lung cancer. With the breath-hold axial 3D VIBE sequence, readers can detect only half of the visible nodules both on pre- and post-contrast images and the post-contrast VIBE sequence did not improve the nodule detection rate for small nodules and non-FDG-avid nodules nor decreased the number of false positive nodules. As of now, the post-contrast VIBE sequence would not be sufficient to overcome the inherent limitation of MR imaging of the lung such as its limited spatial resolution, low contrast resolution of lung parenchymal lesions related to low proton densities and MR artifacts including motion and susceptibility artifacts [26–28].

The low nodule detection rates for non-FDG-avid nodules and nodules < 5 mm of PET/MRI observed in our study corresponds with the results of several previous studies [10, 15, 21] and the number of false-positives varied considerably among the readers. Although small pulmonary nodules do not necessarily indicate metastasis in the presence of an underlying malignancy and missed nodules on PET/MRI may not always cause serious clinical consequences in most cases, we believe that the substantial number of false-negatives as well as false-positives may be a limitation of clinical application of current hybrid PET/MR system as a whole body oncologic imaging study, and would need to be improved in the near future to substitute PET/CT.

There are some limitations to our study. First, we included only a small number of non-lung cancer patients with abdominal and genitourinary tract cancers. Second, the role of PET in the hybrid PET/MRI system was not been fully investigated in our study. Readers interpreted the PET images using the PACS system rather than using a viewer and software specifically designed for PET reading. Third, the nodule detection performances of PET/CT and PET/MRI were not compared in this study as the readers did not perform a nodule detection study using PET/CT. However, this was beyond the scope of our study purpose, which aimed to compare the nodule detection rates of pre- and post-contrast VIBE images within the PET/MRI system.

In conclusion, the addition of post-contrast VIBE sequence to hybrid PET/MR imaging provided no added value in the detection of pulmonary nodules in patients with primary malignancies.
